# In Vitro Analysis of Camellia sinensis Leaf Extract Against Diabetes Mellitus

**DOI:** 10.7759/cureus.62794

**Published:** 2024-06-20

**Authors:** Srimathi B, Priyadharshini R, Selvaraj Jayaraman

**Affiliations:** 1 General Pathology, Saveetha Dental College and Hospitals, Saveetha Institute of Medical and Technical Sciences, Saveetha University, Chennai, IND; 2 Oral and Maxillofacial Pathology, Saveetha Dental College and Hospitals, Saveetha Institute of Medical and Technical Sciences, Saveetha University, Chennai, IND; 3 Centre of Molecular Medicine and Diagnostics (COMManD) Department of Biochemistry, Saveetha Dental College and Hospitals, Saveetha Institute of Medical and Technical Sciences, Saveetha University, Chennai, IND

**Keywords:** inhibition, enzyme, camelia, diabetes, diabetes type 2, invitro, docking study, herbal drugs

## Abstract

Introduction: Diabetes mellitus (DM) poses a significant global health challenge, with its prevalence steadily increasing. Natural compounds derived from plants have garnered attention for their potential therapeutic effects in managing this metabolic disorder. *Camellia sinensis*, commonly known as tea, is rich in bioactive compounds exhibiting various pharmacological properties. This study investigates the potential anti-diabetic activity of *C. sinensis* leaf extract through *in vitro *analysis.

Materials and methods: *Camella sinensis *leaf extract was prepared by grinding the plant's leaf into a powder, mixing it with distilled water, and heating. The antidiabetic activity was assessed through α-Amylase and α-Glucosidase inhibitory assays, employing varying concentrations of the plant extract. Molecular docking analysis utilized Autodock 1.5.6 software (The Scripps Research Institute, California, US) to predict ligand-receptor interactions, guiding subsequent experimental validation.

Result: *Camella sinensis *leaf extract exhibited high phenolic content, suggesting potential in managing hyperglycemia. Tannins may aid glucose absorption and inhibit adipogenesis, making them promising for non-insulin-dependent DM (NIDDM). Terpenoids, with antioxidant activity, inhibit advanced glycation. Saponins and steroids were absent. Molecular docking revealed residues like IR, IRS1, and AS160 with significant impact on α-Amylase and α-Glucosidase, comparable to metformin.

Conclusion: The findings of this study highlight the promising potential of *C. sinensis *leaf extract in managing hyperglycemia associated with DM. The high phenolic content aids in glucose regulation. Specifically, the presence of tannins suggests a potential role in modulating glucose absorption and inhibiting adipogenesis, which could be particularly beneficial for individuals with NIDDM. These findings provide valuable insights into the molecular mechanisms underlying the potential therapeutic efficacy of *C. sinensis* leaf extract against DM, paving the way for further research and development of novel therapeutic interventions in diabetes management.

## Introduction

Diabetes mellitus (DM) is a chronic illness characterized by improper metabolism of proteins, lipids, and carbohydrates. It is one of the most common non-communicable diseases worldwide, affecting people of all ages [1]. Most forms of diabetes are chronic (lifelong), and all forms are manageable with medications and/or lifestyle changes [2]. Diabetes occurs when blood sugar (glucose) levels are too high due to insufficient insulin production by the pancreas or improper body response to insulin. According to the International Diabetes Federation, the prevalence of diabetes, which stood at 10.5% in 2021, is expected to rise to 11.3% by 2030 and 12.2% by 2040 [3].

Insulin binds directly to its receptors on the cell's plasma membranes to initiate action. These receptors are present in all cells, with the highest density in hepatic and adipocyte cells [4]. Metformin is a multifaceted medication with several molecular mechanisms and sites of action. It acts on the liver to lower glucose production and on the gut to increase glucose utilization, increase glycogen-like peptide-1, and alter the microbiome. The enzyme α-Glucosidase, found in the brush border of the small intestine, breaks down disaccharides and starches. Within the digestive system, α-Amylase dissolves the internal α-1, 4-glycosidic bonds that bind starch to glucose and maltose [5]. Pancreatic amylase is produced in the small intestine by the pancreas, while salivary glands contain amylase. The α-Amylase helps determine blood glucose levels by improving the digestion of starch and disaccharides. Delaying glucose absorption by inhibiting enzymes such as α-Glucosidase and α-Amylase is a therapeutic method for treating DM [6]. The postprandial glucose level rises in diabetic patients due to the breakdown of carbohydrates by these enzymes. Controlling postprandial hyperglycemia and inhibiting these enzymes' activity can decrease diabetes risk [7].

One approach to treating DM involves herbal therapy and dietary supplements, such as *Camellia sinensis *leaf extract, which inhibits the activity of α-Amylase and α-Glucosidase [8]. *C. sinensis*, an evergreen shrub or small tree in the plant family Theaceae, is used to produce tea. The plant has long been used to treat several illnesses, including diabetes, rheumatoid arthritis, bacterial infections, and hyperlipidemia [9]. Studies have shown that compounds in *C. sinensis*, such as kaempferol, lutein, isoquercitrin, epicatechin, quercetin, ellagic acid, and epigallocatechin gallate, can reduce blood sugar, increase insulin secretion, and boost β-cell function [10]. Inhibiting α-Glucosidase and α-Amylase using *C. sinensis* can significantly decrease the post-prandial rise in blood glucose [11]. This strategy can be useful in managing blood glucose levels in type 2 diabetic and borderline patients [12]. This study aims to understand the efficacy of phytochemical compounds derived from *C. sinensis* leaf extract against DM. The evaluation will be conducted using *in vitro* and computational methods to provide valuable insights into the molecular interactions and potential mechanisms of action.

## Materials and methods

Preparation of plant extract 

The leaf of *C. sinensis *plant was acquired from Panchagar, Bangladesh, which lies at 26.33°N latitude and 88.56°E longitude. It was subsequently verified and authenticated by the Botanical Survey of India, receiving the authentication number SVMC/BOT/203/2022-23. The leaf of *C. sinensis* was ground into a powder. An amount of 10g powder was combined with 100ml of distilled water and heated on a mantle for 10-15 minutes. The mixture was filtered to procure the *C. sinensis *extract. 

Antidiabetic activity

α-Amylase Inhibitory Activity

An amount of 50μl of phosphate buffer, 10μl of α-Amylase, and 20μl of plant extracts at different concentrations (0.1-0.5 mg/ml) were preincubated at 200°C for 20 minutes. Then, 20μl of 1% starch was added and incubated at 370°C for 30 minutes. An amount of 100μl 3,5-Dinitrosalicylic acid (DNS) color reagent was added and boiled for 10 minutes. Optical density was measured at 540nm. Acarbose was used as standard.

% inhibition = (1-AS/AC) × 100

α-Glucosidase Inhibitory Activity

An amount of 50μl of phosphate buffer, 10μl of α-Glucosidase, and 20μl of plant extract at different concentrations (0.1-0.5 mg/ml) were preincubated at 370°C for 15 minutes. Then, 20μl of p-nitrophenyl glucopyranoside (P-NPG) was added and incubated at 370°C for 20 minutes. The reaction was stopped by adding 50μl of sodium bicarbonate. The absorbance was measured at 405nm. Acarbose was used as standard.

% inhibition = (1-AS/AC) × 100

Molecular docking analysis

A computer method, called molecular docking, is used to forecast the manner and affinity of a small molecule's (ligand's) binding to a target protein or receptor. It is extensively used in researching protein-ligand interactions, virtual screening, and drug discovery. The target protein must first be prepared. The protein structure might be inferred from computational techniques or determined from experimental methods. To prepare the ligand, its 3D structure is either created using molecular modeling software or acquired from experimental sources. It is possible to optimize the ligand to minimize energy. During docking, the ligand is handled like a rigid structure. Docking programs give a score to every docking pose, making it possible to identify ligands with a high affinity. Promising ligands are chosen for additional experimental validation after docking findings are examined and visually appraised. BIOVIA Discovery Studio (Dessault Group, Paris, France) performed the docking analysis in Autodock 1.5.6 software (The Scripps Research Institute, California, US) and the 2D and 3D visualization.

## Results

The phytochemical screening revealed a high presence of phenols, which may contribute to managing hyperglycemia, Tannins can increase the absorption of glucose and suppress adipogenesis, making them promising medications for the management of non-insulin-dependent DM (NIDDM). Terpenoids have antioxidant activity and inhibit the formation of advanced glycation. Saponins and steroids are not present in *C. sinensis*. Enzyme activity may be decreased by phytochemical substances (phenol, tannins, and terpenoids), suggesting a high potential for antioxidant activity. The findings imply that *C. sinensis* has strong anti-glycating properties. It may be because polyphenolic chemicals are present (Table 1).

**Table 1:** Qualitative phytochemical screening of C. sinensis

S. No.	Phytochemical Compounds	Presence (+)/Absence (-)
1	Phenol	++++
2	Saponins	-
3	Tannins	++
4	Steroids	-
5	Terpenoids	+

**Figure 1:**
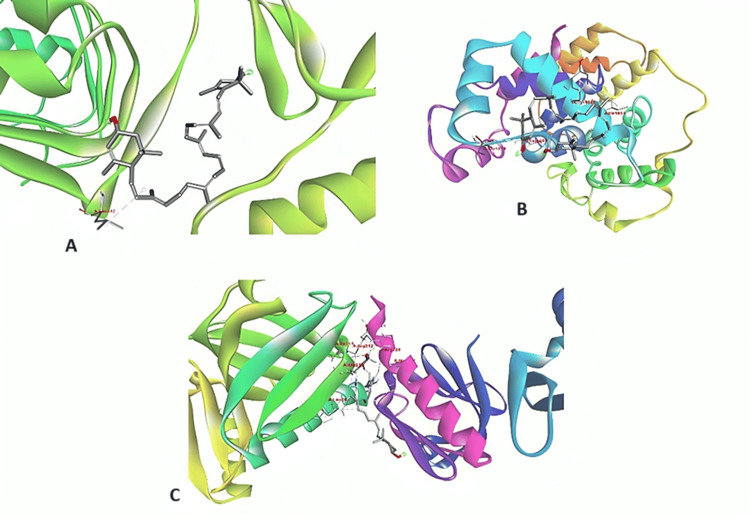
3D structure visualized using Biovia Discovery Studio. Representation of the docked orientation of α-Amylase and α-Glucosidase and their corresponding counterparts: A) IR spectroscopic analysis of protein, B) IRS1, and C) AS160 IR: Infrared; IRS1: Insulin receptor substrate 1; AS160: Akt substrate

The study examines the significant effect of inhibiting α-Amylase and α-Glucosidase of three proteins (IR, IRS1, and AS160). Notably, AS160 exhibits the highest impact among the extracts compared to a standard drug reference (metformin), which is also statistically significant. One of the most useful tools for examining a protein's active site and for understanding and elucidating the binding relationships between ligands and the target protein is molecular docking. The three key protein compounds chosen were IR, IRS1, and AS160 for the molecular research of α-Amylase and α-Glucosidase inhibition. Through individual chemical interactions with the targeted pancreatic α-Amylase and α-Glucosidase, comparative research was conducted. The results showed that the compounds from AS160 had the highest docking score, nearly identical to the standard reference medication metformin (Table 2) (Figure 1).

**Table 2:** Molecular docking analysis results using PyRx software

S. No.	Drug	Protein	Binding Energy (kcal/mol)	No. of H bonds Involved	Amino Acid Residues
1	Lutein (CID 5281243)	IR	-6.1	1	LEU342
2	Lutein (CID 5281243	IRS1	-5.1	2	ARG258; MET25
3	Lutein (CID 5281243)	AS160	-4.9	2	PRO1081; MET 1056

**Figure 2:**
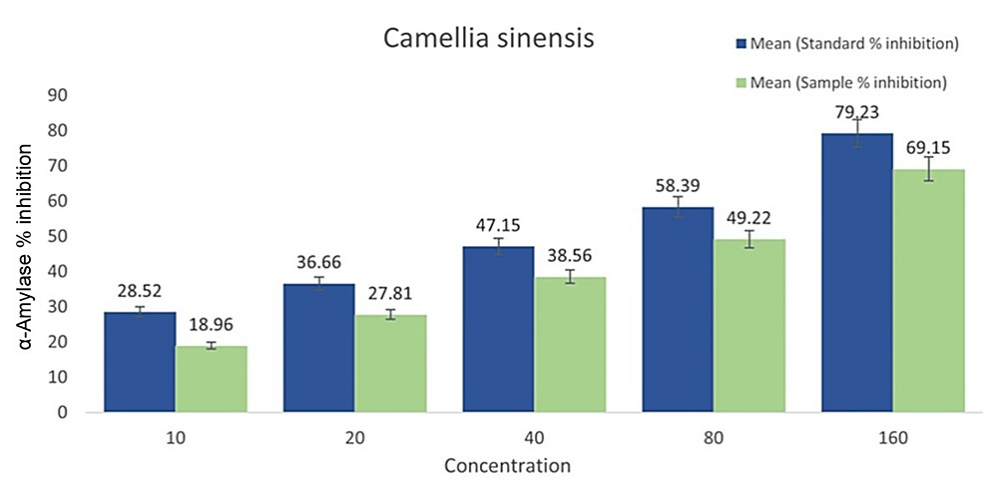
The α-Amylase inhibitory activity of C. sinensis extract with standard antidiabetic medications to benchmark potential clinical relevance In this α-Amylase inhibition, the standard drug is metformin and the sample is *C. sinensis*. The percentage of α-Amylase inhibition increased with the increase in concentration density. Between 80 to 160μg/mL concentrations is the highest inhibitory rate, which shows antidiabetic activity.

Values represent the α-Amylase enzyme inhibitory activities of *C. sinensis*, with higher values indicating higher inhibition. SE (sample) inhibited α-Amylase at concentrations of approximately 1.9µg mL−1, 2.7µg mL−1, 3.6µg mL−1, 4.7µg mL−1, and 6.4µg mL−1, respectively. These inhibitory effects of the extracts were comparable to those of the positive control and the standard medication (metformin), which exhibited concentrations of approximately 2.4µg mL−1, 3.5µg mL−1, 4.3µg mL−1, 5.6µg mL−1, and 7.5µg mL−1, respectively (Figure 2).

**Figure 3:**
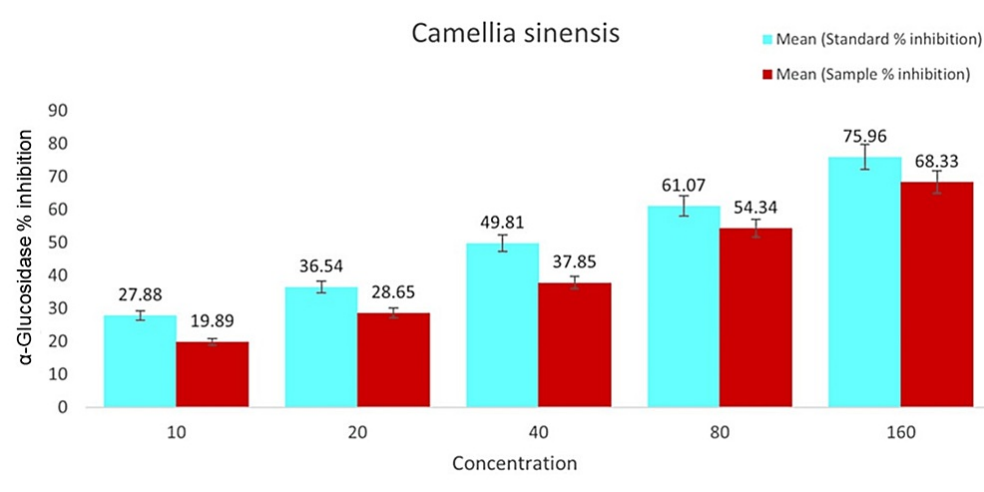
Leaf extract of C. sinensis has inhibitory effects on α-Glucosidase. The correlation between the concentration groups of *C. sinensis* and the level of α-Amylase inhibition is shown in the reference chart. The *C. sinensis* leaf extract concentration groups are shown on the x-axis. The α-Glucosidase % inhibition is shown on the y-axis. The *C. sinensis* leaf extract is shown in blue and the common medication metformin is red. For each bar, the mean ± SD value is indicated.

The inhibitory effects of *C. sinensis* on the α-Glucosidase enzyme were expressed through values, with higher values indicating higher inhibition. Regarding α-Glucosidase, SE (sample) exhibited inhibitory activity at approximately 0.9µg mL−1, 1.7µg mL−1, 2.8µg mL−1, 3.6µg mL−1, and 5.9µg mL−1, respectively. These inhibitory effects of the extracts were comparable to those of the positive control, followed by the standard medication metformin that showed concentrations of approximately 1.8µg mL−1, 2.7µg mL−1, 3.3µg mL−1, 4.9µg mL−1, and 6.3µg mL−1, respectively (Figure 3).

## Discussion

This study provides a detailed overview of the research findings regarding the potential anti-diabetic properties of *C. sinensis* extract. The study focuses on *C. sinensis* chemical composition and its effects on key enzymes involved in glucose metabolism, specifically α-Amylase and α-Glucosidase. Compared to previous research articles, this study corroborates and expands upon the understanding of *C. sinensis* as a potential therapeutic agent for managing hyperglycemia and NIDDM [13]. Identifying phenols, tannins, and terpenoids in *C. sinensis* aligns with existing literature, emphasizing their role in modulating glucose metabolism and exerting antioxidant effects.

The absence of saponins and steroids in *C. sinensis* further distinguishes its chemical profile from other plant sources, reinforcing its unique pharmacological potential. The focus on α-Amylase and α-Glucosidase inhibition adds significant depth to the existing body of research. By examining the inhibitory effects of *C. sinensis* extract on these enzymes, particularly in comparison to the standard drug metformin, the study highlights the potency of *C. sinensis* in modulating carbohydrate digestion and glucose absorption. Notably, the superior inhibitory activity of* C. sinensis*, particularly compounds from AS160, its therapeutic efficacy, and its potential as a natural alternative to conventional medications [14].

Molecular docking analysis provides valuable insights into the binding interactions between *C. sinensis* compounds and target proteins, shedding light on the mechanisms underlying its anti-diabetic effects [15]. The high docking scores observed, comparable to metformin, signify the strong affinity of *C. sinensis* compounds towards the target enzymes, further supporting its therapeutic relevance. The study data demonstrates dose-dependent inhibition of α-Amylase and α-Glucosidase enzymes by *C. sinensis* extract. The concentration-dependent increase in inhibitory activity parallels the standard drug metformin, reaffirming the clinical relevance of *C. sinensis* in diabetes management in correlation with the previous study [16]. Overall, this study on previous research findings provided comprehensive insights into the chemical composition and pharmacological activities of *C. sinensis* extract. The findings underscore its potential as a natural anti-diabetic agent, warranting further investigation into its therapeutic mechanisms and clinical applications.

Limitations

The study predominantly relies on *in vitro* experiments to evaluate the anti-diabetic properties of *C. sinensis* extract. While these assays offer essential preliminary data, they may not fully represent the complex physiological interactions occurring *in vivo*. Future research should address these factors to facilitate the translation of preclinical findings into clinical practice effectively.

## Conclusions

The study revealed the potential of *Camellia sinensis* as a therapeutic agent for managing hyperglycemia and non-insulin-dependent diabetes mellitus. The rich presence of phenols in *C. sinensis* suggests its efficacy in glycemic control, while tannins exhibit promise in enhancing glucose absorption and suppressing adipogenesis. Additionally, terpenoids display antioxidant properties and inhibit advanced glycation, further supporting the anti-glycating potential of *C. sinensis*. Notably, molecular docking analyses reveal that AS160 compounds exhibit significant inhibitory effects on α-Amylase and α-Glucosidase, comparable to the standard drug metformin. It suggests a promising avenue for utilizing *C. sinensis* in diabetes management. The values obtained for α-Amylase and α-Glucosidase inhibition highlight the potency of *C. sinensis* extracts, with inhibitory activities comparable to positive controls and standard medication. Overall, our findings provide compelling evidence for the anti-diabetic properties of *C. sinensis*, driven by its diverse phytochemical composition and potent enzymatic inhibition capabilities.
